# 

**Published:** 1899-01-07

**Authors:** 


					rQSI so SPIT A Ci. ? The standard of highest purity."?Lancet. Jan. 7th, 1899.
CADBURY'S 1UT fel CADBURY'S
ABSOLUTELY PURE.
COOOA ?L OOCOA
wvwwm B> <= -?=? r<m; ii NO DRUOS or alkalies used.
No. 641. Vol. XXY. [ggg?] Saturday,
Jan. 7th, 1899.
(-Sub8onpUonTermBtj pRICE TWOPENCE.

				

